# Toward a Cure: Does Host Immunity Play a Role? 

**DOI:** 10.1128/mSphere.00138-17

**Published:** 2017-04-26

**Authors:** Jielin Zhang, Clyde S. Crumpacker

**Affiliations:** Department of Medicine, Beth Israel Deaconess Medical Center, Harvard Medical School, Boston, Massachusetts, USA; University of Michigan—Ann Arbor

**Keywords:** cure, epigenetic immunity, epigenetics, host immunity, human immunodeficiency virus

## Abstract

Three decades of research on human immunodeficiency virus (HIV) and AIDS reveal that the human body has developed through evolution a genome immune system embodying epigenetic regulation against pathogenic nucleic acid invasion. In HIV infection, this epigenetic regulation plays a cardinal role in HIV RNA production that silences HIV transcription at a molecular (RNA) level, controls viral load at a cellular (biological) level, and governs the viremic stage of AIDS at the clinical (patient) level. Even though the human genome is largely similar among humans and HIV is a single viral species, human hosts show significant differences in viral RNA levels, ranging from cell to organ to individual and expressed as elite controllers, posttreatment controllers, and patients with AIDS. These are signature biomarkers of typical epigenetic regulation whose importance has been shunted aside by interpreting all of AIDS pathogenesis by the known properties of innate and adaptive immunity. We propose that harnessing the host genome immune system, defined as epigenetic immunity, against HIV infection will lead toward a cure.

## Abstract (precis)

The decades of research on HIV and AIDS has contributed to an explosion of knowledge on immune responses. Pivoting on it, we reveal a host genome’s immune system that is defined as epigenetic immunity, utilizing it to protect eukaryotic DNA against the HIV infection to reprogram the patient’s immunity and to develop successful vaccines toward a cure for AIDS.

## INTRODUCTION

Viruses are the most abundant pathogens on earth and infect all species of living organisms, including bacteria, parasites, and humans. The genomes of all complex organisms are composed of DNA, which is a potential target for invasion by viruses and transposable elements. A family of animal viruses known as retroviruses that includes human immunodeficiency virus (HIV) is able to transcribe its RNA genome reversely to DNA and integrates this viral DNA into host DNA to form a provirus during the infection. The viral DNA needs to be integrated into a host DNA for the retrovirus to complete its life cycle. In the absence of proviral integration into host DNA, the outcome is an abortive infection (1).

Viruses can cause worldwide infections called pandemics. Among these global epidemics, HIV and AIDS have drawn great attention, not only for the toll on human lives and on the global economy but also for increasing our understanding of immunity against the retroviral infection. The HIV infection unprecedentedly targets and destroys CD4 T lymphocytes, well-known as the regulators of both the human innate and adaptive immune responses.

After more than 3 decades of research on HIV-AIDS, the field is now moving toward a cure, relying mainly on highly active antiretroviral therapy (HAART) or combination antiretroviral therapy (cART) that can control HIV replication (2, 3) and the ability of the human hematopoietic stem and progenitor cells (HSPC) that not only are resistant to HIV infection (4–7) but also able to replenish all blood cells daily in the human body. The current trend of progress toward a cure, however, appears to miss a fundamental point of how to harness the explosion of knowledge about the immune response that HIV research has generated and how to have antiviral therapy act synergistically with host immunity to reinstitute the patient anti-HIV response and lead to a cure.

Although the development of anti-HIV therapy is a success, patients who receive HAART and markedly block viral replication do not exhibit a cure, that is they do not show a complete reconstitution of their anti-HIV immunity. A second important point pertains to the fact that the most effective remedy to stop viral diseases and cure patients has been immunization or vaccination, but unlike some other viral pandemics, there is no effective vaccine for HIV-AIDS in spite of more than 3 decades of intensive research on this topic. Last, patients with HIV-AIDS reveal different patterns of HIV infection whether treated with HAART or not. These patients include elite controllers, posttreatment controllers, and patients with AIDS, and they can be distinguished by the HIV RNA level in blood that is called the viral load. The HIV RNA level in blood or the viral load is a direct measure of viral replication and defines a patient’s clinical symptoms as well as phenotype.

A significant mechanism to control infectious diseases is the human immune system that controls the replication of the pathogen, and is able to eradicate the pathogens, with or without the aid of antimicrobial drugs (8). Although passive immunotherapies or passive immunization may show a certain efficacy in some patients or macaques, these trends are not likely to cure pandemic infectious diseases. In an era of modern medicine, personalized/precision medicine is being developed to find cures that may enhance the functions of human immunity. One of the approaches is to use the technology that is built on the explosion of knowledge of immune responses that HIV-AIDS research has produced to modulate the host genome’s immune system (8, 9). This technology will utilize the host genome immune system, developed through evolution, to protect our genome from invasion by pathogenic nucleic acids, including that by HIV.

In this opinion article, we hope to begin a discussion toward understanding the fundamentals of how the genome’s immunity or epigenetic immunity protects genomic DNA against retroviral invasion that is underscored by the human intrinsic immune responses. We focus on revealing that epigenetic immunity, in addition to the known innate and adaptive immunities, plays a pivotal role in the control of HIV infection, reprogramming patient immunity, and successful potential AIDS vaccination toward a cure.

### Epigenetic immunity.

Epigenetic immunity is defined as the host genome’s protection system embodying epigenetic regulation. In agreement with findings in plants and other animals, we propose that the human genome’s immune system comprises a selective protective response to pathogenic nucleic acid invasion, which is epitomized by DNA methylation, histone modification, and noncoding RNA (ncRNA) interaction. All three of these components belong to essential epigenetic regulation, entailing the terminology of epigenetic immunity.

Eukaryotes have a genome’s immune system that protects genomic DNA against foreign nucleic acid invasion (8, 9). Current HIV-AIDS research has provided a new infrastructure of knowledge that the human immune system is able to protect the function and integrity of our DNA against pathogenic nucleic acid invasion at the molecular, cell, tissue, organ, and systemic level. Understanding and delineating the underlying mechanisms of anti-HIV immunity, also known as epigenetic immunity, will allow us to make monumental progress toward a cure for AIDS and also cancer, because it is a genome’s immunity (1–9). Here we will examine and focus on how epigenetic immunity contributes to an HIV-AIDS cure.

First and foremost, let us refresh the basic knowledge of epigenetics, a study of changes in organisms caused by modification of gene expression rather than a change in the DNA sequence and the heritable nature of this modification. Epigenetics provides answers to one of the central mysteries of biology, namely, how do cells with the same genetic code take on different phenotypes and identities?

By focusing on HIV infection, AIDS pathogenesis, and working toward a cure, 3 decades of research on HIV-AIDS have established that HIV is a retrovirus and the cause of AIDS. The virus targets the DNA genome of host immune cells, and the expression of HIV RNA in host blood, specifically the CD4 T cells, is the hallmark of HIV infection.

Epigenetic regulation consists of DNA methylation of CpG dinucleotides, covalent modification of histones in chromosomes, and the interplay of ncRNAs in the genome. All three key epigenetic regulatory features are revealed in the studies of HIV-AIDS pathogenesis, specifically DNA methylation of HIV promoters (10, 11), chromatin remodeling on HIV proviral function (12, 13), and ncRNAs, particularly microRNA (miRNA), in inhibiting HIV RNA expression (14, 15), which encompass the epigenome realm. In other words, the host has developed, through evolution, epigenetic regulations against foreign nucleic acid invasion that has played the cardinal roles against HIV infection.

Awareness of the importance of epigenomic regulation has been shunted aside by interpreting AIDS pathogenesis, so far, only by attention to the known innate and adaptive immune responses, instead of to measure or assess the DNA wide immune responses that silence or block HIV RNA expression in individuals at the molecular, cell, tissue, organ, and systemic level. The epigenetic responses, including the DNA methylation on the HIV long terminal repeat (LTR) promoter, ncRNAs on the viral RNA activity, and histone modifications on the host chromatin remodeling, all play a commanding role on the HIV RNA transcription that is signified by the viral load. How could the epigenetic immunity, the fundamental mechanism of silencing HIV RNA expression become an unsung hero, unnoticed, in the arena of anti-HIV immunity and of reconstitution of anti-HIV responses?

Traditionally, the integrated human immune response has been divided into two components: innate and adaptive (or acquired) immunity. It is generally recognized that the human defense system against microbial infection consists of three components: an anatomic and physiologic barrier, innate immunity, and adaptive immunity. As is clearly shown in HIV infection and AIDS pathogenesis, a failure in any one of these systems greatly increases the susceptibility for HIV infection, involving enhanced integration of HIV proviral DNA into the host genome and specifically, expression of HIV RNA, detected as the viral load in the blood of patients that results in a clinical diagnosis of AIDS.

The host immune response to control viral RNA expression is primarily by epigenetic regulation (8–15). Toward the goal of an HIV cure, the role of epigenetic response is as important as that of the innate and adaptive immune responses, and this is also true in interpreting HIV-AIDS pathogenesis. Without an in-depth understanding of the mechanism of epigenetic regulation and its responsive protective functions against HIV infection at both the proviral DNA and its RNA level, we will not succeed in efforts to block HIV-RNA transcription and eradicate the HIV viral load.

With our current knowledge, the HIV RNA level is the signature of HIV infection and designated as the viral load, viremia, and diagnostic criterion. At the molecular level, HIV RNA is regulated by the host transcriptional machinery. In the context of HAART consisting of no anti-Tat drugs so far and with or without HAART, the host epigenetic regulation still plays a cardinal role in controlling HIV RNA production. This has been shown to consist of silencing of HIV RNA transcription at the molecular (RNA) level, controlling viral load at the cellular (biological) level, and governing viremia and the stage of AIDS at the clinical (patient) level.

**FIG 1:**
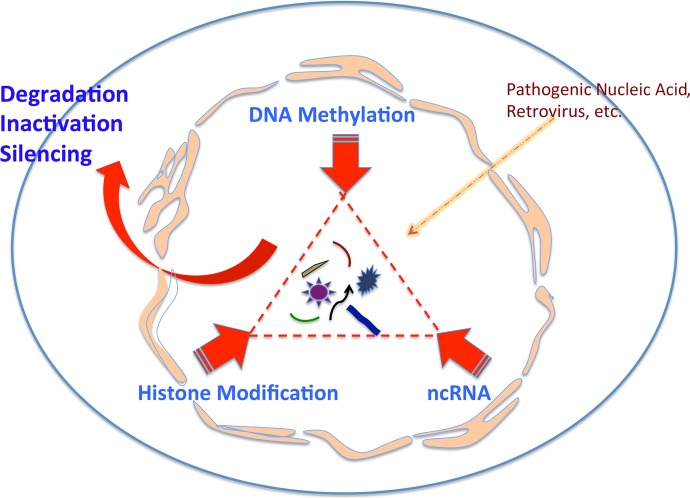
Three core factors of epigenetic immunity—the host genome’s immunity. The host protects its DNA from foreign nucleic acid invasion via an epigenetic mechanism, which consists of DNA methylation, histone acetylation/methylation, and noncoding RNA (ncRNA) activity. Each works independently but cohesively with the other two, protecting the structure and function of the host genome, ranging from heterochromatin, euchromatin, up to RNA transcription and silencing.

Furthermore, human beings show great diversity, and with a similar human genomic DNA against the single species of HIV, different hosts show significant differences in HIV RNA transcriptional levels. This is a hallmark of typical epigenetic regulation, epitomizing a host epigenetic control to silence HIV proviral DNA gene expression at the RNA transcriptional level. In RNA transcription from HIV DNA, the integration or lodge site, i.e., the status of DNA sequence and its chromatin topological or spatial feature, govern the activity of an HIV provirus (Fig. 1). This is in addition to the known innate and adaptive immune responses regarding HIV infection and AIDS pathogenesis. Hence, it is the host epigenetic immunity that shuts off HIV RNA genome production and plays a primary role in all HIV patients, who exhibit different levels of viral loads and receive different diagnoses as elite controllers, posttreatment controllers, and patients with AIDS.

### Discussion.

Epigenetic immunity stems from our 3 decades of research on HIV-AIDS, and this research has contributed to a tsunami of knowledge on human immune responses. In today’s era of personalized medicine, the goal remains to determine the mechanisms where epigenetic immunity contributes to an HIV-AIDS cure. We hope to start the discussion from two aspects that conflate—epigenetic immunity controls the HIV RNA expression, i.e., the viral load, and this governs HIV-AIDS therapy and prevention.

First, the progress employing bench to bedside to desk (*in silico*) studies, namely, of DNA methylation, histone modification, and ncRNA function, has shown that epigenetic immunity silences transcription of HIV RNA and blocks HIV infection by remodeling the HIV DNA, inactivating the HIV promoter, and interfering with viral genomic RNA (8, 10–20). Further in-depth investigations, however, are required to elucidate the essential component, circuit, and pathway that comprise the mechanism of epigenetic immunity, which execute the power of prevention and treatment of immune diseases of not only AIDS but also cancer, via this host intrinsic immune response. Now we outline for a cross-field, multidisciplinary investigation.

### Nuclear membrane.

Through evolution, eukaryotes have the anatomic structure of a nuclear membrane to guard and enclose their DNA genome. The reason is plainly clear: protecting the genomic DNA protects the kernel of human life, and changing DNA expression changes a human life. The role of a nuclear membrane in epigenetic immunity, however, needs to be further explored. In addition to the future in-depth study on HIV entry via penetrating the nuclear membrane with the viral preintegration complex, how epigenetic immunity exerts its protective response against the pathogenic nucleic acid invasion versus cultivates symbiosis with the DNA of mitochondria (mtDNA), human endogenous retroviruses (HERVs), or exosomes, etc., requires a multidisciplinary study (21, 22). We believe the nuclear membrane is the first barrier and a hub that organizes input and output epigenetic immune responses fighting against the pathogenic nucleic acids while courting for the compliant ones, such as the mtDNA, etc.

### In and out circuit.

The human immune system can be viewed as an analogy to the nervous system, having sensor receptor, signal transduction, central control, and effector activity. Epigenetic immunity is the immunity that protects the DNA genome, and its sensor detection to effector execution is, not surprising, like the known innate and adaptive immunity, but beyond that, it exhibits a complexity and efficiency that have surpassed known innate and adaptive immunity. Studies of HIV-AIDS have unveiled several molecules, machineries, and cells whose functions closely relate to the protection of host genomic DNA at the levels of HIV entry, integration, and transcription, and after further study, that not only can be applied in prevention but also therapy.

Some of these are new, and others are known cellular factors with newly discovered functions against HIV infection in a cell-specific manner; these factors include TRIM5α, APOBEC3G, SAMHD1, etc. p21^Cip1Waf1Sdi1^ (p21), a known cell cycle checkpoint protein with functions resisting HIV infection in HSPC, networks in epigenetic regulation via long ncRNA (lincRNA)-p21, belongs to the second group (5, 18). Further study on who is who, meaning who is the sensor or the connector or the effector, remains to be delineated to connect the dots and unravel the circuit.

Moreover, the recently observed adaptive changes in natural killer (NK) cells enabling these cells to have functions between innate and adaptive immunity are reported (23, 24) to show that immune responses to environmental factors, such as viruses, are in line with recent evidence suggesting that human immune systems as a whole vary more as a consequence of epigenetic influences than genetic influences. In the context of NK cells working against viral nucleic acid invasion of the host DNA, the function of NK cells in patient immunity to HIV, and the stem cell properties of memory CD4 T cells, it is imperative that investigators harness expertise in virology, immunology, and molecular biology to make cross-field efforts to connect the dots and delineate the circuits that maneuver a sophisticated, protective epigenetic network toward an HIV-AIDS cure.

This will include, but not be limited to, deciphering the roles of Toll-like receptors (Toll-like receptor 3, 7 or 8, and 9), interferon (alpha, beta, and gamma interferon), deoxynucleoside triphosphate (dNTP) pools, etc. The central control of epigenetic immunity is, of course, the chromatin remodeling embodying DNA methylation, histone modification, and ncRNA function. The input and output pathways of epigenetic immunity, however, from the molecule to cell, to tissue, to a systemic level, are crying out to be defined, filled, and targeted toward an HIV-AIDS prevention, treatment, and cure.

### Epigenetic immune memory.

The epigenetic changes on a host DNA genome, i.e., DNA methylation, histone modification, and ncRNA function, are heritable (4–26). By definition, immunity against HIV via epigenetic responses, also known as epigenetic immunity, is also heritable, shown by the memory CD4 T-cell responses against HIV in elite controllers, posttreatment controllers, and patients with AIDS in spite of shown different degrees of anti-HIV immunity.

Epigenetic immune memory is a transcriptional memory that has been revealed in T cells. The active chromatin modifications are retained at the gene transcriptional level, but they are kept in a silent state and can be rapidly reactivated when required to ensure appropriate immune responses upon receiving environmental “triggering,” embodying a native T cell clonal formation against a specific antigen.

Although no statistically significant results are reported regarding the three epigenetic immune parameters versus the viral load, i.e., the HIV RNA level, it is not a surprise to learn that the studies thus far have demonstrated that epigenetic immunity correlates with the plasticity of anti-HIV immune response and AIDS pathogenesis in HIV patients (5–26). Epigenetic immune memory, such as that of memory CD4 T cells and NK cells, plays the fundamental roles in HIV-AIDS prevention and treatment, the former related to the AIDS vaccine development including therapeutic vaccines, and the latter regarding reinstitution, or more accurately, transcriptional alteration/reprogramming of patient immunity against HIV at the immune effector cell level.

Specifically, against HIV, the quintessential power of epigenetic immunity includes the potency of transcriptional memory or epigenetic immune memory. This immune memory results in a selected effector cell phenotype, filtration, propagation, and perpetuation to exert a reprogrammed, antigen-specific epigenetic immune control. Vaccine development, either prophylactic or therapeutic, stems from the taproot of epigenetic immunity, protects not only the host DNA genome from microbial nucleic acid invasion but also acts synergistically, strengthens, and reprograms the host epigenetic immunity at the effector cell level, especially the memory CD4 T cells and NK cells (7, 23–26).

### Epigenetic immunity in preventive versus therapeutic application.

Smallpox vaccination was developed before we understood how to harness innate and adaptive immunity to protect a host from smallpox infection. The vaccination or immunization process against viral infections, such as influenza virus and Zika virus, remains to be defined and developed. Differing from other viral pandemics, HIV has challenged both innate and adaptive immune systems via targeting memory CD4 T cells, which leaves us to face, poise, and ponder how we harness or maneuver an ancient immune system that has been developed through eukaryotic evolution to protect our DNA’s function.

**FIG 2:**
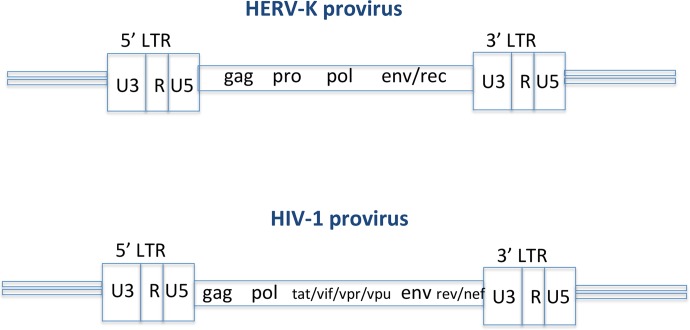
HERVs are close relatives of HIV, many of which have the defective genomes, and are kept silenced permanently in the human genome via epigenetic regulation for generations without causing harm.

In retrospect, epigenetic immunity appears to have played an intrinsic role in silencing the RNA expression of ancient human endogenous retroviruses that are the close relatives of HIV (Fig. 2) (9, 16, 17). It is well-known that HERVs have been present in human genomes for generations, many of them have defective proviral genomes, and there is no viral RNA transcribed or expressed from the HERVs to cause an infectious viral disease. This signals a hallmark effect of epigenetic immunity to protect our genomic functions at the DNA acting level and is a footprint of the integrity of genomic DNA under the sovereign control of the genome’s immunity (8–20).

### Nexus of epigenetic immunity.

The mechanism of genome’s immunity, also known as the exact pathway of epigenetic immunity that controls RNA expression of HERVs, however, requires in-depth study. A nexus of epigenetic immunity between silencing and activating RNA transcription from a foreign nucleic acid requires an overarching epigenetic immune investigation, specifically the manner by which epigenetic immunity smothers HERVs but nurtures DNA of mitochondria, exosome, etc. (21, 22). A multidimensional platform derived by evolution, from an organelle to an infrastructural system, ranging from epigenetic responses to modulations that pinpoint the rudiments of epigenetic immunity, exemplified by the power of memory CD4 T cells and NK cells versus the function of epigenetic immunity against HIV should be investigated at the epigenetic level rather than the genetic level, in contrast to the symbiosis with mtDNA, transposon, and exosome.

Taken together, whether the control of HERV expression occurs via an epigenetic or genetic pathway is an issue correlated with the HIV-AIDS cure. The research of an HIV-AIDS cure, nonetheless, appears to be heading in two directions. One direction is the epigenetic pathway, which is to define how the RNA of HERVs is shut off in humans that belongs to the study of the mechanism of epigenetic immunity. The other direction is the genetic pathway, which is to sequence the DNA of memory CD4 T cells and NK cells or that around HERVs to show the difference.

We believe in taking HIV-AIDS as a model to identify how epigenetic immunity can shut off HIV provirus expression, meaning to control the viral RNA, which is also known as controlling viral load expression. Whether the DNA from memory CD4 T cells and NK cells determines the epigenetic immune responses or the epigenetic immune responses protect the host DNA function on gene expression is out of the scope of reprogramming patient anti-HIV immunity toward a cure. Inasmuch as the HIV-AIDS research is in progress, we believe epigenetic immunity, similar to innate and adaptive immunity, is a heritable host immune response to the environment stimuli, specifically against the invasion of pathogenic nucleic acid to our DNA, embodying chromatin remodeling via DNA methylation, histone modification, and ncRNA function, which silences the expression of proviral HIV in a host memory CD4 T cell as well as in other immune cells that have genomic DNA.

The treatment remedy derived from host epigenetic immunity can be in all forms of the genome’s epigenetic regulation, including the DNA sequence that participates or underscores the chromatin remodeling after the HIV infection, showing a signature response that consists of epigenetic immunity. Employing precision/personalized medicine, we modulate epigenetic immunity to treat HIV infection, and the patient DNA sequence embodying epigenetic responses is a part and the root of epigenetic immunity, so long as now no one changes the DNA sequence before understanding the epigenetic immunity. As you do an operation, know anatomy first. Inasmuch as you do stimulation, know the synapse first. If you cut the genome, know the chromatin conformation/configuration first. Because you went ahead to cut the DNA without knowing chromatin remodeling or epigenetic immunity, you would destroy the genome’s immunity—epigenetic immunity.

Last but not least, defining the tipping point between epigenetic immunity and inflammation will lead to exerting immunity while curtailing nonprotective inflammation and immune exhaustion at the RNA level, also known as the preeffector level, to silence viral replication and reduce the cell damage at the threshold of pretranslation (protein), preeffector (cells), or preenzyme (pathway) in a holistic manner (8–20).

Current experimental results compel us to look at host anti-HIV immunity from a new aspect of genomic to epigenetic immunity, which specifically controls RNA transcription and viral load by host epigenetic immunity that leads to an HIV-AIDS cure. If we consider that epigenetic regulation is a mechanism by which the host regulates its gene expression, specifically at the RNA level, and the same mechanism protects the host genome from pathogenic nucleic acid invasion, such as HIV or other retroviral infections, then ignoring the epigenetic mechanism in host immunity against HIV is to ignore a fundamental immune principle in understanding, enhancing, and harnessing the host immunity against HIV and other viral infections (Fig. 1 and 2).

Finally, we suggest that the strategies and funding for an HIV-AIDS cure should focus on connecting all the dots in epigenetic mechanisms that protect the host genome from HIV infection via silencing viral gene expression, specifically HIV RNA expression, i.e., the viral load. By the same token, epigenetic immunity is capable of controlling the activities of latent viral reservoirs. Combining regulation of epigenetic immunity with HAART, nonetheless, results in targeting the RNA-DNA-RNA life cycle of HIV, enforcing selected mutations to occur at both HIV genomes, speeding and ushering HIV in becoming a second HERV that is compromised dramatically by decreasing enzymatic activity, particularly integrase, and RNA transcription from its defective provirus, the viral DNA (25). A precision regulation of host epigenetic immunity either silences HIV expression or lives symbiotically with HIV or coerces HIV to become another HERV, thereby eliminating the viral reservoirs. The gist of epigenetic immune therapy is to exert the patient epigenetic immunity at both the host genomic structure (DNA remodeling) and DNA function (RNA expression) levels against a microbial infection.

Simply put, epigenetic immunity underpins the mechanism of a patient anti-HIV immune response, playing an indispensable role toward both HIV eradication and AIDS cure. For the development of a vaccine or immunization, epigenetic immunity plays pivotal roles not only on the modification of vaccine DNA but also on the efficiency of vaccine RNA expression, specifically on CD4 T cell-helped innate and adaptive immunity, via immune memory, self-renewal, and effector cell differentiation.

The basis of epigenetic immunity should be further explored, delineated, and utilized from DNA, RNA, cell, tissue, organ to a systemic (patient) manner in order to unravel the epigenetic regulatory pathways consisting of host anti-HIV immunity, to recapture the epigenetic response to develop an AIDS vaccine, and to reinstitute the patient epigenetic immunity against HIV toward a cure. Even though it is generally recognized that epigenetics plays an important role in patient immunity against HIV infection, harnessing epigenetic immunity toward a cure for HIV-AIDS is a multidisciplinary task, ranging from virology, immunology, molecular biology, cell biology (stem cell biology), genetics, to epigenetics.

Last, the same principles of epigenetic immunity, the genome’s immunity, apply to the cancer treatment and cancer vaccine development, aligning with the development of immune therapy based on epigenetic regulation in this field. In particular, research on the compounds acting as inhibitors of DNA methylation reveals a treatment shift in cancer immunotherapy. The newly established mechanism “tricks” cancer cells to behave as virus-infected cells by triggering an HERV “mimicry” state to induce the antiviral response, which includes the activation of interferon-responsive genes and that of programmed cell death (apoptosis) in suppressing and clearing the viral infection, to be an effective therapeutic approach to malignant cells. Untangling the diversity of the genome’s immune system will ultimately lead to a cure of immune maladies, including but not limited to AIDS and cancer.

We hope that by focusing on understanding the basis of epigenetic immunity, we can further explore, understand, and utilize host immunity toward a cure for AIDS and cancer. Let us recapture the epigenetic responses in developing vaccines, in reinstituting or reprogramming the patient’s epigenetic immunity, including the immune memory, against AIDS and cancer that are at the forefront of an array of immune diseases requiring a cure.
